# Defining a Modular Signalling Network from the Fly Interactome

**DOI:** 10.1186/1752-0509-2-45

**Published:** 2008-05-19

**Authors:** Anaïs Baudot, Jean-Baptiste Angelelli, Alain Guénoche, Bernard Jacq, Christine Brun

**Affiliations:** 1 Institut de Biologie du Développement de Marseille-Luminy (IBDML), UMR6216, CNRS/Université de la Méditerranée, Marseille, France; 2 Institut de Mathématiques de Luminy (IML), UMR6206, CNRS/Université de la Méditerranée, Marseille, France; 3 Spanish National Cancer Research Centre (CNIO), Structural Biology and Biocomputing, Melchor Fernández Almagro, 3 E-28029 Madrid, Spain

## Abstract

**Background:**

Signalling pathways relay information by transmitting signals from cell surface receptors to intracellular effectors that eventually activate the transcription of target genes. Since signalling pathways involve several types of molecular interactions including protein-protein interactions, we postulated that investigating their organization in the context of the global protein-protein interaction network could provide a new integrated view of signalling mechanisms.

**Results:**

Using a graph-theory based method to analyse the fly protein-protein interaction network, we found that each signalling pathway is organized in two to three different signalling modules. These modules contain canonical proteins of the signalling pathways, known regulators as well as other proteins thereby predicted to participate to the signalling mechanisms. Connections between the signalling modules are prominent as compared to the other network's modules and interactions within and between signalling modules are among the more central routes of the interaction network.

**Conclusion:**

Altogether, these modules form an interactome sub-network devoted to signalling with particular topological properties: modularity, density and centrality. This finding reflects the integration of the signalling system into cell functioning and its important role connecting and coordinating different biological processes at the level of the interactome.

## Background

'Interactomes' are novel biological entities that correspond, ideally and formally, to the complete set of interactions existing between all the macromolecules of an organism [1]. Currently, the available interactomes are primarily formed by protein-protein interaction (PPIs) networks in which the interactions have been experimentally obtained either from high throughput experiments (such as large-scale two hybrid screens and affinity purifications/mass spectrometry [2-12]) or by different types of low-scale experiments. Despite the fact that interactomes are far from being complete, the current PPI maps (for yeast, worm, fly and human) form large intricate networks [13-16].

Concurrently to the deciphering of the interactomes, bioinformatics methods allowing their analysis have been developed. Since interaction networks are represented by complex graphs in which nodes correspond to proteins and edges to their interactions, a number of these methods have been grounded on principles deriving from graph partitioning theory such as the search for interaction-dense regions [17,18], shortest paths in the graph [19], graph disconnection according to edge betweenness [20,21], the sharing of interactors [22,23] or a combination thereof [24] (see [25,26] for review). All these algorithms partition the interaction network into sub-graphs. Among those, the PRODISTIN method that we previously proposed [22] allows the functional classification of the proteins through the computation of a distance reflecting the sharing of interactors between each possible protein pair. As previously demonstrated, proteins participating to the same cellular processes are clustered by the method into the same PRODISTIN classes [22,27].

Signalling consists of multiple sequential events which relay information by transmitting signals from cell surface receptors to intracellular effectors that eventually activate the transcription of target genes. These events are promoted by specific interactions between signalling molecules (proteins, lipids, ions) among which the more prominent and numerous are the protein-protein ones. Essentially due to the temporal dynamics of signal transduction and to the experimental choices often made for investigation (mainly genetics), the signalling mechanisms have long been perceived and described as distinct and isolated linear cascades of reactions, namely the signalling pathways. Nowadays, this vision is progressively changing with the discovery of important crosstalks between pathways [28,29] and the assumption that unknown crosstalks should be responsible for the difficulty to predict output states for particular pathways [29,30]. Finally, the discovery of large numbers of new participants to well known signalling pathways in metazoans, resulting from novel investigations using functional genomics and proteomics methods [31-39], is widening the signalling space [40].

We have taken advantages of our recent efforts 1) in participating to the deciphering [5] and the manual curation of the Drosophila interactome [41] and 2) in developing PRODISTIN, an interactome analysis method [22,27], to investigate the topological organization of the signalling pathways when embedded within a global PPI network. This may predict the participation of unforeseen actors to the pathways and provide an integrated view of the signalling mechanisms by assessing the existence of important interactions between them.

Thus, we have applied the PRODISTIN method to a high quality Drosophila interactome. We established and analyzed the functional classification of the proteins participating to 9 canonical signalling pathways and identified 12 classes which potentially correspond to 12 functional modules. From the detailed analysis of these modules, their composition and interconnections, it appears that the linear perception of the signalling pathways does not resist to a global interactome analysis. Rather, our work delineates a highly modular and interconnected signalling network showing a central and plausibly organizational position within the global interactome.

## Results

### Functional classification from the protein-protein interaction network: a bi- to tri-partite organization of the signalling pathways based on the sharing of interactors

In Drosophila, at least eleven pathways are crucial for signalling in development and adult cell physiology, namely the Wingless (WG), Hedgehog (HH), Notch (N), Decapentaplegic (TGF), Janus Kinases and Signal Transducers and Activators of Transcription (JAK-STAT), Sevenless (SEV), Torso (TOR), Epidermal Growth Factor Receptor (EGFR), Insulin (INS), Toll (TOL) and Fibroblast Growth Factor (FGF) pathways. These pathways have been chosen for the subsequent analysis and according to our curation of the literature (see Methods for details, Additional file 1), each of them transmits external signals through a cascade of reactions mediated by a ten of canonical proteins (10.63 on average). Given that these proteins are parts of a larger PPI network, studying the structure of these signalling pathways with an interactome perspective may bring new insights not only into their composition and possible regulation but also their integration into cell functioning.

For this purpose, a high confidence Drosophila interactome composed of 2894 binary protein-protein interactions involving 2939 proteins (see Methods) was analyzed with the PRODISTIN method [22,27]. Briefly, this interactome analysis method first calculates a functional distance between each possible pairs of proteins in the interaction network with regard to the number of interactors they share (in order to reduce the weight of spurious interactions in the computation, proteins must have a connectivity k ≥ 3 to be considered further); the distance values are then clustered leading to a classification tree which is subsequently subdivided into formal classes, using the tree topology and the functional annotations of the proteins (see Methods and [22] for detailed explanations and statistical assessments).

**Figure 1:**
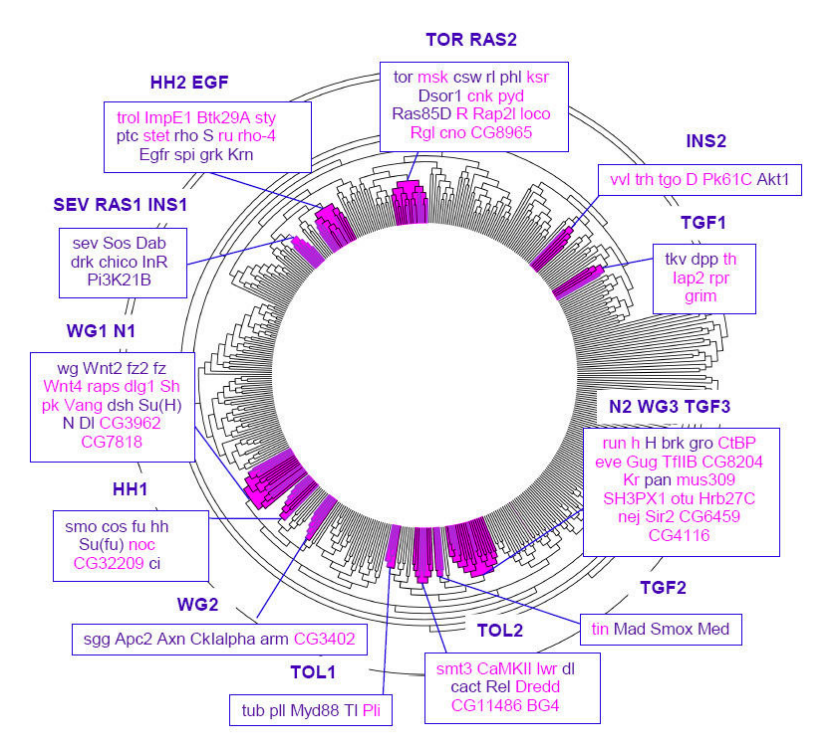
**The Prodistin classification tree and the signalling PRODISTIN classes**. The Drosophila PRODISTIN classification tree contains 472 Drosophila proteins. Protein names have been omitted for clarity. PRODISTIN classes containing signalling canonical proteins are coloured in magenta in the tree. Purple branches carry canonical proteins. Classes are named and numbered according to the signalling pathway's proteins they contain. When proteins from several pathways are present in the same class, the names of the different pathways are juxtaposed. For sake of clarity, the RAS cascade is clearly identified as such despite we didn't consider it as a pathway on its own. Details of the class proteins are shown in boxes using Flybase symbols for genes: canonical proteins are purple, non-canonical ones are magenta.

In the present work, we obtained a classification tree containing 472 proteins (Figure 1) among which 58 are canonical components of 9 out of the 11 signalling pathways cited above. The FGF and JAK-STAT pathways were not further investigated because 80 to 100% of their canonical proteins are so poorly connected in the high quality interactome that they are not classified. On average, 68% of the canonical signalling proteins initially taken into consideration are present in the classification tree (Figure 1).

Then, in order to annotate this tree following the PRODISTIN procedure, classes of proteins involved in the same cellular function(s) were defined according to the GO Biological Process ontology. Among the PRODISTIN classes containing less than 20 proteins (which are nested into larger ones since the method uses a tree representation and Gene Ontology displays a hierarchical organization), those containing at least one canonical protein have been chosen to be further investigated. Twelve such classes have been identified, containing 56/58 of the canonical proteins present in the tree (Figure 1, Additional file 2). On average, these PRODISTIN classes contain 9.75 proteins among which 48% are canonical proteins from the signalling pathways. All these classes but one are annotated with a GO Biological Process term related to 'Signal Transduction', or are nested within a larger class annotated with such a term (by nature, a nested class inherits the annotations of all its parent classes) (Additional file 2). Noticeably, the 'Signal transduction'-related annotation of almost all these classes is supported by the best p-value (calculated as the over-representation of the considered GO term in the class compared with the tree [27]) (Additional file 2), reinforcing the proposal that the proteins belonging to these classes are signalling actors.

We then investigated the detailed distribution of the canonical proteins by analyzing the 12 PRODISTIN classes. Whereas one could have anticipated the clustering of the proteins participating to the same signalling pathway into one single class, it strikingly turned out that without any exception for the 9 considered pathways, the canonical proteins were distributed into 2 or 3 classes per pathway (2.33 on average). Since the PRODISTIN method clusters proteins sharing common interactors [22], this result means that the canonical proteins of a particular pathway belonging to a same class share more common interactors between them and with the other non-canonical proteins of the same class than with the other canonical proteins of the same pathway found in other classes. As a control, ten randomization experiments in which protein names have been randomly assigned to tree branches (see Methods) showed that this clustering pattern could not have happened by chance since the canonical proteins of a pathway are then found distributed among 5.75 (± 0.6) classes on average.

As a conclusion, the analysis based on the sharing of interactors overall suggests a bi- to tri-partite organization of each fly signalling pathway within the interactome.

### The distribution of GO annotations among PRODISTIN classes reflects the polarity of signal transduction: toward the notion of 'signalling modules'

**Figure 2:**
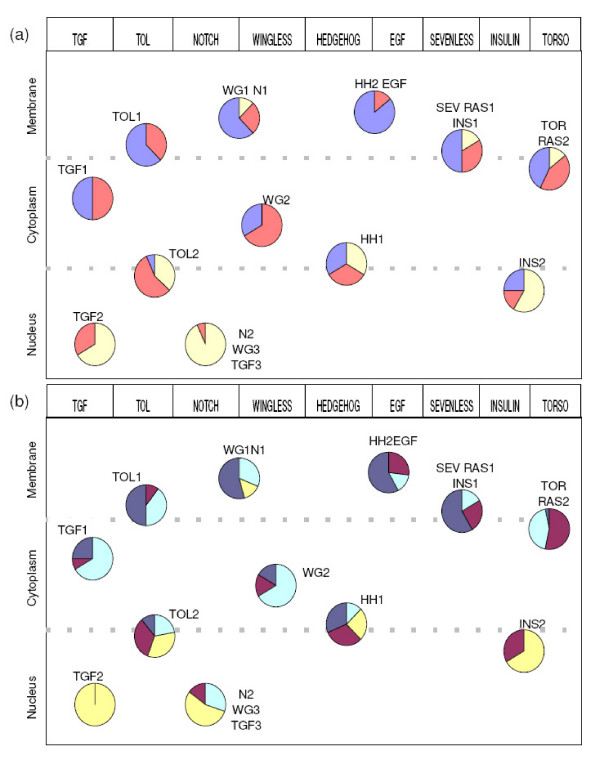
**Gene Ontology annotations of the signalling modules**. The 12 classes identified by the PRODISTIN method are represented as pie charts. They show the proportion of proteins annotated with the terms: Nucleus (wheat), Cytoplasm (salmon) and Membrane/Extracellular (blue) for the Cellular Component ontology **(a) **and Receptor/Ligand (dark blue), Kinase/Hydrolase (dark red), DNA binding proteins (yellow), Others (turquoise) for the Molecular Function ontology **(b)**. For visualization sake, the pie charts have been ordered along the horizontal axis according to the signalling pathways they correspond to, and along the vertical axis according to their main subcellular localization.

For a better understanding of the functional significance of this bi- to tri-partite organization of the signalling pathways, we investigated the class composition and the functions of the classified proteins based on Gene Ontology annotations [42]. Using the 'Cellular Component' and the 'Molecular Function' ontologies, we investigated the repartition of protein localizations and molecular functions in each PRODISTIN class (see Methods for details, Figure 2a and 2b). In addition, we verified that the GO term corresponding to the main sub-cellular localization of each PRODISTIN class was statistically over-represented among the annotations of all the proteins of the class as compared to the fly proteome using the statistical tool of GOToolBox [43] (Additional file 2).

Qualitatively, when considering the main localization of the classes containing the canonical proteins of a given pathway, the sub-cellular polarity of the pathway is perceptible. Indeed, these proteins are subdivided into classes mainly membrane or membrane/cytoplasm-located, and classes mainly cytoplasmic or cytoplasm/nucleus-located (Figure 2a). This partition of the canonical proteins in classes of different main sub-cellular localizations is also corroborated by the distribution of the Molecular Function annotations found within the classes (Figure 2b). Indeed, proteins acting at the same level of a signalling pathway through their interactions, such as ligands and receptors, are clustered in the same class. In other words, when the proteins of a particular pathway are considered, although the dynamic aspect of signalling is not taken into account for this analysis, the 'Molecular Function' and the 'Cellular Component' annotations of the clustered proteins reflect the polarity of signal transduction (Figure 2b). Taken all together, these results lead us to propose that the identified PRODISTIN classes may correspond to 'signalling modules' defined as groups of signalling proteins acting together through their interactions.

### The composition of the signalling modules reflects close relationships between pathways

Since only 12 modules have been identified and each of the 9 pathways is split into 2 to 3 modules, some modules must necessarily include components belonging to more than one pathway. Indeed, whereas 8 out of 12 signalling modules contain canonical proteins from a single pathway, the 4 others enclose proteins from several pathways (Figure 1). These modules contain proteins from the Wingless and the Notch pathways (class WG1 N1), the Wingless, Notch and TGF pathways (class N2 WG3 TGF3), the Sevenless-Ras and the Insulin pathways (class SEV RAS1 INS1) and the Hedgehog and EGFR pathways (class HH2 EGFR). In 3 out of 4 of these 'mosaic' modules, several canonical proteins of the different pathways interact directly (for instance, Notch (N) from the eponymic pathway interacts with wingless (wg) and dishevelled (dsh) from the Wingless pathway, class WG1 N1) (Additional file 3). These protein-protein interactions have different functional contributions to signalling: those mediating a functional crosstalk which involves information transfer from a pathway to another, such as dsh (from the Wingless pathway) inhibiting Notch signalling through its physical interaction with N [44], those revealing the sharing of a component between pathways, such as the use of groucho (gro) from the Wingless pathway as a co-repressor in Notch and TGF signalling [45,46] and those for which no functional role is known so far (detailed in Additional file 3).

The fourth mosaic module, HH2 EGFR, suggests a potential link between the Hedgehog and the EGFR pathways since patched (ptc), the receptor of the Hedgehog pathway, is classified with the membrane/cytoplasmic part of the EGFR cascade (Figure 1). This co-classification is not due to a direct interaction between canonical proteins of the pathways but rather by the sharing of interactors. Indeed, the EGFR ligand gurken (grk) and sprouty (sty), a negative regulator of the pathway, share respectively one and four interactors with ptc. Interestingly, two of these interactors (trol and Ppn) are peptidoglycans known to modulate the signal activity by sequestering ligand molecules or by favouring the ligand-receptor interaction, therefore stressing the coherence of the classification.

Finally, the fact that the HH2 EGFR and the SEV RAS1 INS1 classes contain the membrane proteins of four signalling pathways known to use lipid rafts for signal transduction [47] is also remarkable and reinforces the functional consistency of the modules found by the analysis.

Overall, these results show that although the analysis is anchored on separate linear cascades (represented by their canonical proteins), the co-classification of some of their components by the PRODISTIN method reveals their close functional relationships.

### Signalling modules contain other proteins related to signalling: validations and predictions

The PRODISTIN method clusters proteins participating in the same cellular process by grouping proteins that share interactors. The obtained clusters then provide a mean to identify potential new players in given cellular processes based on their co-classification with proteins clearly involved in these processes [22]. On average, half of the proteins contained in the signalling modules are not canonical proteins. This accounts for 61 proteins out of 117 proteins contained within the signalling modules. Although these 61 proteins are candidates to participate in signalling, our results obtained on a high quality interactome may be dependent on its relative small size. Indeed, a large number of available but possibly false interactions have not been incorporated in the high quality interactome and an unknown number of not yet detected but physiological interactions is probably missing. We thus address the robustness of our predictions based on co-classifications by proposing as putative new members or regulators of the signalling pathways only the proteins systematically found clustered with the canonical proteins both in the high quality network and in a larger one, containing almost all available Drosophila interactions (22819 interactions) (see Methods for details). As a result, 45/61 non canonical proteins contained in the signalling modules are robustly clustered with the canonical proteins (Table 1, Additional file 4). Twenty of them correspond to already known regulators of the pathways after literature and Gene Ontology annotation searches, thus validating our approach. In addition and noticeably, 15 other proteins are members of alternate or other pathways. Here again, we observed that a given signalling module may contain canonical proteins from a particular pathway as well as proteins from another pathway. Interestingly, proteins belonging to signalling pathways not chosen to 'anchor' the analysis (such as IMD (= Immune Deficiency) and JNK (= Jun N-terminal kinase)) are classified within signalling modules defined by the 'anchoring' pathways (TGF1 and TOL2 for IMD, HH2 EGFR and TOR RAS2 for JNK). This finding raises the number of classified pathways from 9 to 11, therefore heavily underlining the fact that with a PPI network perspective, signalling pathways are intermingled. The functional consequences of this observation may reside in the integration of the signalling processes and their capacity to rapidly respond to diverse extra-cellular stimuli.

**Table 1:** Functional status of the proteins classified with canonical proteins in each signalling module.

	**R**	**AO**	**P**
**INS2**	Pk61C		
**TGF1**		th	
		Iap2	
		Rpr	
		Grim	
**N2**	run		Gug
**WG3**	h		Kr
**TGF3**	CtBP		
	eve		
	TfIIB		
	nej		
	Sir2		
**TGF2**	tin		
**TOL2**	smt3	Dredd	CaMKII
	lwr	BG4	
**TOL1**	Pli		
**WG2**			CG3402
**HH1**			CG32209
**WG1 N1**		Wnt4	CG3962
		Pk	CG7818
		Vang	
**HH2**	trol	Btk29A	ImpE1
**EGF**	sty		
	stet		
	ru		
	rho-4		
**TOR**	msk	Pyd	Loco
**RAS2**	ksr	R	CG8965
	cnk	Rap2l	
		Rgl	
		Cno	

R, protein already known to regulate the pathway; AO, protein known to be involved in alternate or other pathways; P, protein predicted to be involved in the pathway by this analysis.

### Signalling modules contain new potential actors of the pathways

Finally, among the 45 non-canonical pathway proteins robustly found in the 12 signalling modules, 10 proteins are neither known regulators of the pathways nor members of other pathways (Table 1, Additional file 4). They are thus predicted by the classification to participate to signalling processes. Five of them have been previously described as involved in other biological processes (Additional file 4) but their domain composition is compatible with a possible implication in signalling. The five others (described below) did not have any Gene Ontology Biological Process annotations in FlyBase at the time of the work but arguments from the literature available for 4 of them suggest their potential role as components, regulators or effectors of the signalling pathways in Drosophila.

#### Hedgehog pathway

CG32209/Serpentin is found in the HH1 module, containing the hedgehog (hh), smoothened (smo), costal-2 (cos), fused (fu), Suppressor of fused (Su(fu)) and cubitus interruptus (ci) proteins. It has been recently involved in late tracheal development [48,49]. Its possible involvement in the Hedgehog pathway is suggested by several lines of evidence. First, CG32209 is interacting with the receptor patched (ptc) and the transcription factor ci *via *two different domains with a high confidence score [5]. Second, CG32209 contains a Low Density Lipoprotein (LDL)-like receptor domain and hedgehog is a lipidated molecule. From a genetic point of view, a transposon insertion into CG32209 is presumably lethal [50] and the gene belongs to a complementation group rescuing the *jaft *mutation, identified in an enhancer/suppressor genetic screen designed to characterize novel components of the Hedgehog pathway [51].

#### Notch pathway

CG3962/Keap1 is found in the signalling module WG1 N1 containing the Notch (N) and Delta (Dl) proteins and interacts with Dl. Taken together with the facts that 1) the human ortholog KEAP1 is an adaptor protein regulating steady-state levels of the transcription factor NRF2 in response to oxidative stress [52] and 2) the accumulation of reactive oxygen species in mammalian cells was recently shown to occur following disruption of Notch signalling [53], CG3962 may play a role in the Notch pathway.

#### Ras pathway

In agreement with its classification in the signalling module containing the cytoplasmic part of the Ras cascade (TOR RAS2), CG8965, which contains two RA (Ras-associated) domains, was recently proposed to represent a Ras effector candidate based on its interactors [5].

#### Wingless pathway

CG3402 belongs to the WG2 module containing the cytoplamic part of the Wingless pathway (armadillo (arm), shaggy (sgg), Axin (Axn), Adenomatous polyposis coli tumor suppressor homolog 2 (Apc2), Casein Kinase I alpha (CkIalpha) and CG3402). The protein contains a single PDZ domain – usually found in diverse signalling proteins – of 123 amino-acids, evolutionarily conserved from arthropods to humans, which is interacting with arm with a very high confidence score [6]. The interaction is conserved throughout the evolution since recorded in *C. elegans *[4], *M. musculus *and *H. sapiens *[54]. Intriguingly, CG3402 also interacts with an arrestin-like protein of unknown function (CG18745) [6], whereas arrestins are regulators of (G-protein)-coupled receptor signalling [55], and G-proteins are involved in both the canonical and the non-canonical Wingless pathways [56]. Finally, the facts that the mammalian ortholog is implicated in (G-protein)-coupled receptor signalling [57] and inhibits the transcriptional activity of β-catenin [54], strongly suggest that CG3402 may represent a new player of the Drosophila Wingless pathway.

### Interactions between modules define the signalling network

**Figure 3:**
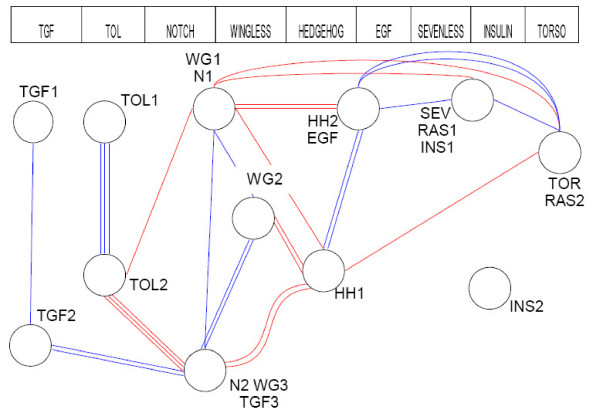
**Interactions between signalling modules**. The 12 signalling modules identified by the PRODISTIN method are represented as circles. The lines between circles represent the 30 protein-protein interactions linking the signalling modules. The 16 blue lines and the 14 red lines correspond to protein-protein interactions linking different modules in the same pathway and in different pathways respectively. The INS2 module is disconnected from the other signalling modules since only direct interactions between modules are here represented.

The composition of the signalling modules defined by our interactome analysis method shows that signalling pathways are closely intertwined. This observation naturally prompted us to next investigate the interactions between signalling modules. An interaction linking any protein of a signalling module to a protein belonging to another signalling module is considered here as a link between those modules. We then differentiate intra-pathway links *i.e *between the different modules assigned to a same signalling pathway, excluding links within a module, and inter-pathways links between modules of different signalling pathways. Among the 30 links connecting the 12 signalling modules together (Additional file 5), 53% correspond to intra-pathway links whereas the other 47% are inter-pathways links (Figure 3). Thus, there is almost no numerical difference between the 'classical pattern' of interactions of the signalling modules in the spirit of the linear cascade and the links between signalling modules of different signalling pathways. This observation holds when considering, the interaction pattern of each protein of the signalling modules in the larger dataset (42% intra-pathways links, 58% inter-pathways links). Given that almost all inter-pathways links (13 out of the 14, Figure 3) involve canonical signalling proteins (Additional file 5), these results are thus extending our previous observation that signalling pathways are intertwined within modules, to a higher organizational level corresponding to the links between signalling modules. The functional contributions of these particular interactions to signalling are diverse and are detailed in Additional file 5.

Is the interaction pattern between signalling modules different from the one between other PRODISTIN classes composing the interactome? To test this, we calculated the density of interactions linking the modules as the number of existing interactions between modules compared to the number of possible ones (see Methods for details). We found that whereas the average density of interactions within modules does not show any important discrepancy between the signalling modules and the other classes (0.39 ± 0.17 vs. 0.35 ± 0.16 respectively), the average density between them shows variations (Table 2). Indeed, the average density of interactions between signalling modules is 6 times higher than between non-signalling PRODISTIN classes. It is also 2 times higher than between all the classes composing the interactome (Table 2). Finally, it is 3 times higher than the average density calculated between 12 classes picked randomly, taken as a control (see Methods for details).

**Table 2:** Interaction densities between modules.

Dataset	Between signalling modules (× 10^-3^)	Between all modules except signalling (× 10^-3^)	Between all modules (× 10^-3^)	Between modules taken randomly (× 10^-3^)
High-quality	5.15	0.86	2.72	1.6
Large	7.73	4.69	4.73	4.02

These results are obtained on a high quality but relatively reduced interactome. Consequently, they might be influenced by the size of the interactome studied. We re-calculated the density measures between modules after considering the interaction patterns of each protein in the larger dataset. We showed that again, the average density of interactions between the signalling modules is still 1.6 times higher than between all classes and twice as high as between random classes (Table 2).

A numerical bias towards signalling interactions in the studied datasets may have influenced the density results. As a matter of fact, whereas the mean connectivity of the proteins of the high quality PPI network is almost 2, the canonical proteins are connected to 5.17 interactors on average. Are the signalling proteins genuinely more connected than others datasets' proteins because of their intrinsic signalling function or is it explained by the fact that a larger number of interactions is known for signalling proteins due to their extensive investigation? This question has been addressed by analyzing the number of interactors identified for both types of proteins (signalling canonical vs. others) in a same set of experiments, therefore in a context devoid of any bias. We compared the number of interactors identified for 13 of the canonical proteins when tested as baits in the LS-Y2H screen of Formstecher *et al*. [5] to the number of interactors found for the 86 other baits tested in the same screen. In these conditions, canonical proteins are 1.6 times more connected than other proteins (9.15 interactors on average vs. 5.89 for non canonical and 5.97 for randomly picked proteins, Additional file 6). In addition, whereas 7.6% of the interactions of the canonical baits involve another canonical protein, only 1.4% of the interactions of non canonical proteins do. Therefore, these results provide support to the fact that the observed higher density of links between signalling modules is not due to a bias towards signalling interactions but rather to the natural higher connectivity of the signalling proteins and their propensity to interact with other proteins involved in signalling.

Taken together, these results thus suggest that we here define a modular sub-network of the interactome devoted to signalling into which interactions between signalling modules are prominent.

### The signalling network lies centrally in the interactome

**Figure 4:**
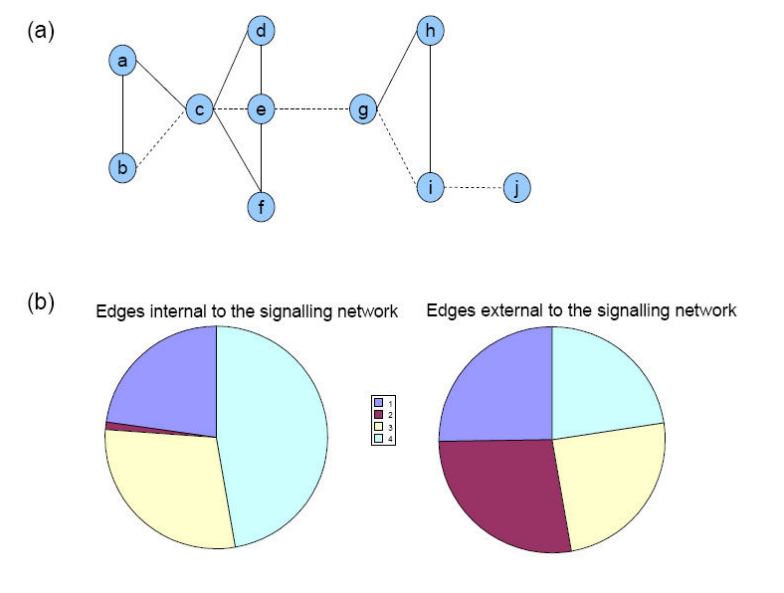
**Analysis of edge-betweenness distribution in the Drosophila PPI network**. (a) A theoretical example of a network between 10 vertices linked by 13 edges (both solid and dashed lines) for the 'shortest path' and the 'edge-betweenness' (EB) definitions. The shortest path between two nodes represents the path for which the number of edges is minimized: the shortest path between nodes b and j is represented by the path in dashed lines. The betweenness of an edge represents the number of shortest paths running through it. For instance, the EB value of the edge i-j corresponds to the number of nodes in the rest of the graph; the EB value of the edge a-b is 1, because only the shortest path between the nodes a and b runs through this edge; the EB value of the edge e-g is the higher of the graph since all shortest paths between nodes located on the left part on one hand, and on the right part on the other hand, must run along it. (b) Piecharts showing the repartition of the subsets of edges internal (left) and external (right) to the signalling network among the 4 interquatile intervals of the EB values distribution. For each subset of edges, the interquartile intervals (labelled from 1 to 4) are indicated by the same colors (chart in the middle).

The betweenness of an edge is the number of shortest paths between all possible pairs of nodes that run along it (Figure 4A). This graph topological feature has been used to define community structures in networks and to partition them [20,21]. It is assumed that when communities are loosely connected by few edges, all shortest paths between node pairs must run through those, therefore leading to a high edge-betweenness (EB) value. Consistently, when the EB values are calculated for all the Drosophila network's edges, signalling modules are linked by high betweenness edges (19167 paths/edge on average vs. 5304 paths/edge on average for all network's edges).

Edge-betweenness is also interpreted as a measure of network centrality [58,59]. Indeed, edges with high EB values have been proposed to control the communications between network's nodes and to contribute to the cohesiveness of the network. In this respect, signalling processes are expected to contain a large number of edges with a high EB value. In order to test this possibility, we studied the distribution of the EB values on the complete network. Then, considering separately two subsets of edges, internal and external to the signalling network, we determined their repartitions among each of the 4 interquartile intervals of the EB values distribution (see Methods for details) (Figure 4, Additional file 7). Whereas the edges external to the signalling network are evenly distributed between the four intervals of the distribution (Figure 4b, right), the edges internal to the signalling network exhibit a non-uniform distribution with two striking features. On one hand, half of them (47%, p < 10^-12^) belongs to the fourth interval of the distribution. This interval contains the higher EB values of the distribution, corresponding to the more central routes of the network according to the EB definition. On the other hand, the internal edges are almost absent of the second interval of the distribution (p < 10^-22^). This interval is essentially populated by the outward edges of the network, connecting proteins solely linked to the connected component *via *one interaction. Therefore, the edges of the signalling network are excluded from the periphery of the network and the number of shortest paths running along them is significantly high. The signalling network thus appears to lie centrally in the interactome based on the EB calculation. This result may reflect the important role of the signalling network in connecting and coordinating the different biological processes and the integration of the signalling system into cell functioning.

## Discussion

By computing the Drosophila interactome, we identified a modular signalling network lying centrally in the network after its topological properties. The deciphering of this highly connected sub-network underlines the topological importance of the interactions between signalling modules for the coherence of the interactome. The study also contributes to identify potential new players in Drosophila signalling.

It is important to note that the PPI network is static and does not contain any spatio-temporal information. Therefore, our conclusions are drawn from the analysis of 'a long-exposure photograph' [60] of the interactions between proteins, *i.e*. the set of all possible interactions in all possible biological contexts.

### Computation of the Drosophila interactions: quality assessment

Protein-protein interaction networks have been often suspected to contain erroneous interactions, to be incomplete and biased towards certain type of interactions. For these reasons, our conclusions have been drawn from the analysis of a high quality interaction dataset in order to minimize the weight of potential false positive interactions. In addition, in order to build robust predictions and conclusions, we have reinforced and validated them on a larger dataset containing almost all the currently known Drosophila PPIs. This step insures that the results obtained on the high quality dataset are not sensitive to missing interactions and robust to potential false positive ones. The PRODISTIN method has also been largely statistically assessed for robustness against the presence of false interactions in a previous study [22].

### Functional modules devoted to signalling

The present analysis identified a signalling network formed by 12 groups of proteins organized around signalling proteins, that we assimilated to 'signalling modules'. Although several different definitions of 'modules' are found in the literature, from static to dynamic (for examples [61-63]), it is however admitted that they form groups of molecules, possibly evolutionary conserved, involved in the same pathway, the same protein complex or the same cellular process. In our experiments as well as in others [17,19,22], modules identified by the computation of interaction networks are generally more than molecular complexes. They may contain proteins belonging to complexes as well as regulatory proteins and/or proteins involved in the same cellular process through interactions. These proteins thus do not necessarily act and bind each other at the same location and time in the cell. As a consequence, a common sub-cellular localization of the proteins of a same functional module is not mandatorily expected since, as known for numbers of signalling proteins, they may shuttle and translocate from one sub-cellular compartment to another to perform their function(s). Indeed, we observed that half of the Drosophila signalling modules are distributed between two sub-cellular localizations and the other half between 3 (Figure 2a).

The identification of functional modules allowed us to predict the participation of 10 potential new actors to Drosophila signalling. None of them was found in the high-throughput RNAi screens recently performed on Drosophila signalling pathways [31-35]. This lack of overlap is probably due to the fact that RNAi screens identify regulators of the pathways which may act not only through protein-protein interactions but also through protein and other molecules (nucleic acids, lipids, ions) direct or indirect interactions.

### Modularity and signalling

**Figure 5:**
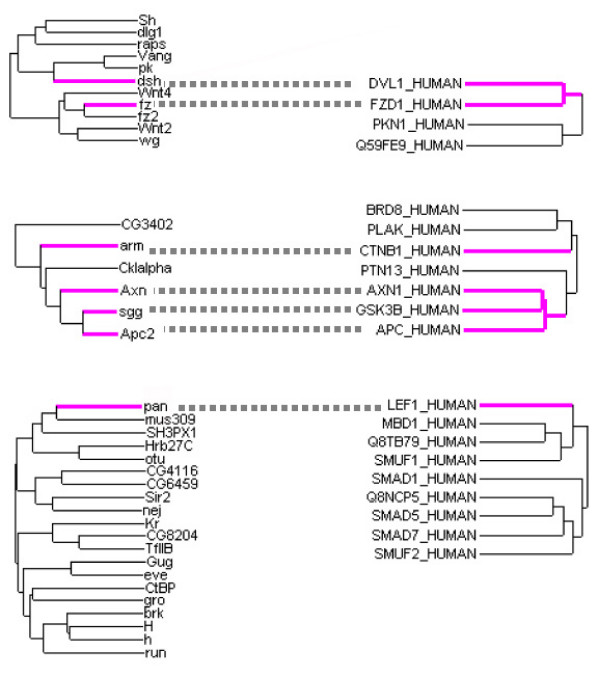
**The human functional homologs of the Drosophila Wingless pathway are also distributed in 3 signalling modules**. The 3 Drosophila PRODISTIN classes containing the canonical proteins (magenta) of the Wingless pathway (left side) are presented in front of the 3 subtrees containing their functional homologs in the human classification tree (right side).

By anchoring our analysis on the currently known signalling pathways herein considered as models [64], and by computing the PPI network they belong to, we showed their systematic bi- to tri-partite modular organization. Here, we generalize an observation made on one pathway of a unicellular eukaryote [19] to the major signalling pathways of a metazoan organism. Moreover, preliminary results obtained on the human interactome (Baudot, Brun, Jacq, unpublished) confirm this organization, at least for the Wnt pathway. Indeed, the human functional homologs of the canonical proteins of the Drosophila Wingless pathway are also distributed between 3 signalling modules (Figure 5). Moreover, the internal composition of each signalling module appears to be conserved throughout evolution.

In theory, the modular organization of the biological networks has been proposed to favour and even ensure the insulation necessary to the correct accomplishment of certain cellular process on one hand, and the connections needed to integrate information from multiple sources on the other hand [61]. Remarkably, these properties constitute two important needs of the signalling process. So, how does this modular organization of the signalling pathways delineated by computational means fit with the functional requirements and principles of the signalling mechanisms? In mammalian cells, the Ras/MAPK signalling takes place in several subcellular compartments (plasma membrane, endosomes, endoplasmic reticulum, Golgi apparatus and mitochondria) [65]. It was shown experimentally that the sensitivity to an input of a MAPK module downstream of Ras – composed of RAF, ERK, MEK and KSR – is determined by its spatial localization [66]. In Drosophila, we found the Ras pathway organized in 2 modules: SEV RAS1 INS1, formed by the membrane-bound proteins of the pathway and TOR RAS2, formed by kinases and the scaffold protein. Interestingly, the latter recapitulates the tested mammalian MAPK module. Indeed, it contains the Drosophila counterparts of the mammalian proteins belonging to the tested module (namely phl for RAF, Dsor1 for MEK and ksr for KSR). Taken together, these results lead to the proposal that the organization of the signalling pathways into different modules may provide the flexibility necessary to the functioning of the same signalling pathway in different spatial, cellular or developmental contexts, aiming probably at increasing the output repertoire complexity.

### High density and high betweenness of edges: two topological features specific to the signalling network

Two graph features, density and betweenness of edges, allowed us to delineate the signalling sub-network from the rest of the network. These topological characteristics – a high density of edges linking the modules and the high number of shortest paths running through the signalling network's edges – reveal the central position of the signalling network within the global interactome. Hence, the role of the signalling mechanisms in connecting and coordinating the diverse cellular processes is here underlined by graph features.

Edge-betweenness is a common concept in graph analysis. However, the question of its exact functional biological meaning remains open. The signalling network encompasses a large number of edges with high EB values. This leads to envisage that this could reflect, like in social networks [58], an information flow in spite of the fact that edges in PPI networks are not directed. This last statement agrees with the recent proposal of Yu and colleagues that nodes linked by such edges correspond to the dynamic components of the PPI network [59].

## Conclusion

We propose here a systems-level analysis of signal transduction from a protein-protein network point of view. Overall, our results reflect the integration of the signalling system into cell functioning and its important role in connecting and coordinating the different biological processes at the level of the interactome.

## Methods

### Protein-protein interactions datasets

The high quality protein-protein interactions dataset is composed of 2894 binary interactions between 2939 proteins. It was created by joining 970 interactions extracted from literature (deposited in the Intact database [41]), to the interactions identified in two LS-Y2H screens with a high confidence score: 584 interactions from Formstecher *et al*. (with A, B or C PBS scores) [5] (i.e. 25% of the interactions identified in this screen) and 1395 interactions from Giot *et al*. (score > 0,8) [6] (i.e. 7% of the interactions identified in this screen).

The large dataset contains 22819 interactions and is intended to represent our present view of the Drosophila interactome (probably largely incomplete and with an unknown proportion of false positive interactions). It contains the 970 interactions extracted from the literature and the complete sets of interactions identified in the two LS-Y2H screens cited above, depleted of 7% of their interactions respectively. These 7% correspond to the interactions with the lowest confidence scores in each of the screens. Finally, 1654 interactions from Stanyon *et al*. [67] were also added.

Furthermore, and with the aim of limiting the effect of false positive interactions, each interaction was weighted depending on its reliability. Taken into account that interactions are taken from different sources and therefore are provided with confidence scores calculated differently, we determined the weights as follows:

- 2894 interactions coming from the high quality dataset are weighted with the maximum value, 1;

- interactions from Giot *et al*. [6] given by the authors with a probability score between 0.5 and 0.8 and interactions from Stanyon *et al*. [67] are weighted with an intermediate value of 0.5;

- interactions from Giot *et al*. [6] given by the authors with a probability score below 0.5 as well as Formstecher *et al*. [5]interactions with a PBS D score are weighted with a minimum value of 0.1.

### PRODISTIN functional classification

#### Applying the Prodistin method on the high quality protein-protein interactions dataset

We used the PRODISTIN method [22] through the Prodistin Web Site [27]. Starting with a list of binary interactions, only proteins involved in at least three binary interactions are selected for further classification in order to reduce the weight of spurious interactions. The server then computes the Czekanowski-Dice distance between all possible pairs of proteins (for details, see [22]). The obtained values are subsequently clustered using the BioNJ algorithm [68], leading to a classification tree containing 472 proteins. PRODISTIN functional classes are identified as the largest possible sub-tree composed of at least 3 proteins sharing the same Gene Ontology (February 29th, 2006 version) Biological Process annotations [42] and representing at least 50% of the class members for which an annotation is available. For each protein, the complete hierarchy of annotation terms is considered (child and all parents). Given the large number of annotations for proteins, the majority of PRODISTIN classes are nested within other PRODISTIN classes.

The result of the computation is then visualized as a coloured classification tree using an integrated TreeDyn module [69] as a tree viewer.

The method has formerly led to the prediction of the cellular function of uncharacterized yeast proteins [22] and the definition of a scale of functional divergence for yeast paralogs based on PPIs [13]. It has also been recently used through its automated version [27] to explore a predicted genetic interaction network of *C. elegans *[70].

#### Canonical proteins and PRODISTIN classes identification

Canonical proteins (see Additional file 1) were identified from the literature as the main actors of the 'canonical signaling pathways', defined according to STKE as 'idealized or generalized pathways that represent common properties of a particular signalling module or pathway' [71].

Signalling classes are identified as classes containing less than 20 proteins and containing at least one canonical protein. Other PRODISTIN classes are identified as non-overlapping classes of the same size.

#### PRODISTIN method on the large weighted protein-protein interactions dataset

Aiming at increasing the efficiency and the reliability of the PRODISTIN classification for large networks, we used in the computation of the distance, the interactions' confidence scores provided in the different large-scale experiments. These confidence scores can be considered as probabilities of interactions and were used to weight the edges of the interaction graph. In order to enable the PRODISTIN method to be applied to weighted networks, we propose to extend the formula of the Czekanowski-Dice distance as follows [72]. This distance was used to cluster graphs Γ = (*X, E*) where X is a set of *n *vertices and E a set of *m *edges. The original distance formula is:

$$D(x,y)=\frac{\left|\Delta \bar{\Gamma }(x),\bar{\Gamma }(y)\right|}{\left|\bar{\Gamma }(x)\right|+\left|\bar{\Gamma }(y)\right|}$$

where Δ is the symmetric difference between two sets, $\bar{\Gamma }\left(x\right)$ the neighborhood of *x *extended by *x *itself: $\bar{\Gamma }(x)=\left\{x\right\}\cup \left\{y|(x,y)\in E\right\}$ = {*x*} ∪ {*y*|(*x*, *y*) ∈ *E*}.

The new distance between each pair (*x, y*) is computed in a graph Γ = (*X, E, W*) where W is the weight function *W *: *X *× *X *→ [0,1].

$$D_{w}\left(x,y\right)=(1-w(x,y))\times \frac{S(x,y)+2}{T(x,y)+2-2w(x,y)}+w(x,y)\times \frac{S(x,y)}{T(x,y)+4-2w(x,y)}$$

where:

$$S(x,y)=\sum_{s\in \Gamma (x)\cup \Gamma (y)}\left|w(x,s)-w(y,s)\right|\text{ and }T(x,y)=\sum_{s\in \Gamma \left(x\right)\cup \Gamma \left(y\right)}w(x,s)+w(y,s)$$

with *Y *= Γ (*x*) ⋃ Γ (*y*)

We then applied the PRODISTIN method based on the new distance formula for weighted edges, on a list of 22819 binary weighted interactions. The obtained classification tree contained 3975 proteins. Since more than 40% of the tree proteins are of unknown function, only a small number of PRODISTIN classes were identified in this tree. Thus, for comparison purposes, we here considered sub-trees (based on topology only) instead of PRODISTIN classes when necessary.

### GO annotation analysis

The Gene Ontology (version February 29th, 2006) Cellular Component annotations of each signalling module protein have been slimmed to a list of annotations comprising the 4 following terms: Membrane or Extracellular, Cytoplasm, Nucleus and Other. Similarly, the GO Molecular Function annotations have also been simplified to a list of 4 terms: Receptor or Ligand, Kinase or Hydrolase, Transcription Factor and Others. Pie charts shown in Figure 2 represent the proportion of the different slimmed annotations for modules' proteins. For multi-annotated proteins, each annotation is given an equal weight such that the sum of the weights is equal to 1.

### Density calculation

The density of links *D*_*i *_within a class *X*_1 _and *D*_*e *_between two classes *X*_1 _and *X*_2 _in a graph Γ = (*X, E*) are defined by the two formulas:

$$D_{i}=\frac{\left|(x,y)\in E\;st\;x,y\in X_{1}\right|}{\left|X_{1}\right|\left(\left|X_{1}\right|-1\right)/2}\text{ and }D_{e}=\frac{\left|(x,y)\in E\;st\;x\in X_{1}\;y\in X_{2}\right|}{\left|X_{1}\right|\left|X_{2}\right|}$$

The density of links within a class *D*_*i *_corresponds to the number of observed links between the class' vertices divided by the number of possible links.

The density of links between a set of more than 2 classes is calculated as the sum of densities between all pairs of classes divided by the number of possible pairs between classes.

### Edge betweenness calculation and distribution study

The total number of shortest paths between all pairs of vertices in a graph that run through a given edge defines its edge-betweenness. If there is more than one shortest path between a pair of vertices, each path is given an equal weight such as the total weight of all paths is equal to 1 [21]. The EB value has been calculated for the 2061 edges of the interactome. Then, we considered 2 subsets of edges:

- the 188 edges linking the proteins belonging to the signalling network, hereafter called 'internal' to the signalling network

- the other 1873 edges connecting either two proteins outside of the signalling network or one inside and one outside, hereafter called 'external' to the signalling network.

For each subset, the repartition of the corresponding EB values is represented according to the interquartile intervals of the initial EB value distribution as piecharts (Figure 4b).

P-values are calculated using the hypergeometric distribution.

## Authors' contributions

AB performed all bioinformatic analysis, participated to the design of the study and to the manuscript's writing. JBA and AG elaborated the classification method for weighted graphs. BJ was involved in data acquisition, conceived of the study, and participated in its design. CB conceived of the study, participated in its design and coordination, and wrote the manuscript. All authors read and approved the final manuscript.

## Supplementary Material

Additional file 1 **Canonical signalling proteins**. List of proteins selected as canonical proteins (see Methods) for each of the 10 signalling pathways. Protein names correspond to FlyBase gene symbols. Click here for file

Additional file 2 **Signalling modules details**. Class name and class members, class annotations and p-values for each annotation; annotation(s) of the larger class and number of proteins in the larger class when the signalling classes are nested into larger ones; p-value of the slimmed GO Cellular Component annotation which is over-represented in each class, computed with the GOStat statistical tool of GOToolBox [33]. Click here for file

Additional file 3 **Interactions between canonical pathways**. List of protein-protein interactions between canonical proteins from different signalling pathways belonging to a same signalling module. For each interaction, the literature source and a short description are given, when available. Click here for file

Additional file 4 **Functional status and Gene Ontology annotations of the proteins classified with canonical proteins in each signalling module**. The functional status of each protein found in signalling modules (except canonical proteins) is defined as R when the protein is already known to regulate the pathway; AO, when the protein is known to be involved in alternate or other pathways; P, when the protein is predicted to be involved in the pathway by this analysis. For each protein, all available GO annotations in the Biological Process, Molecular Function and Cellular Component ontologies are given. GO annotations are printed in blue if they correspond to a predicted annotation (inferred from Sequence Similarity or Inferred from Electronic Annotation) and not to an experimentally proven function. Click here for file

Additional file 5 **Protein-protein interactions between the different signalling modules**. The 30 links connecting the signalling modules are detailed. The source column indicates whether the interaction has been identified using small scale approaches or large scale two-hybrid screens. In the latter case, the PMID and the reference of the paper have a purple background. Click here for file

Additional file 6 Comparison between the connectivity of signalling proteins vs. others. Click here for file

Additional file 7 Analysis of edge-betweenness distribution in the large Drosophila PPI network. Click here for file
